# Reliability and validity of the script concordance test for postgraduate students of general practice

**DOI:** 10.1080/13814788.2017.1358709

**Published:** 2017-08-18

**Authors:** Julie Subra, Bruno Chicoulaa, André Stillmunkès, Pierre Mesthé, Stéphane Oustric, Marie-Eve Rougé Bugat

**Affiliations:** ^a^ University Department of General Practice, Toulouse-Rangueil Faculty of Medicine Toulouse France; ^b^ Inserm U1027, Faculty of Medicine Toulouse France

**Keywords:** Script concordance test, general practice, clinical reasoning, postgraduate students

## Abstract

**Background:** The script concordance test (SCT) is a validated method of examining students’ clinical reasoning. Medical students’ professional skills are assessed during their postgraduate years as they study for a specialist qualification in general practice. However, no specific provision is made for assessing their clinical reasoning during their postgraduate study.

**Objective:** The aim was to demonstrate the reliability and validity of the SCT in general practice and to determine if this tool could be used to assess medical students’ progress in acquiring clinical reasoning.

**Methods:** A 135-question SCT was administered to postgraduate medical students at the beginning of their first year of specialized training in general practice, and then every six months throughout their three-year training, as well as to a reference panel of 20 expert general practitioners. For score calculation, we used the combined scoring method as the calculator made available by the University of Montreal’s School of Medicine in Canada. For the validity, student’ scores were compared with experts, *p* <.05 was considered statistically significant.

**Results:** Ninety students completed all six assessments. The experts’ mean score (76.7/100) was significantly higher than the students’ score across all assessments (*p* <.001), with a Cronbach’s alpha value of over 0.65 for all assessments.

**Conclusion:** The SCT was found to be reliable and capable of discriminating between students and experts, demonstrating that this test is a valid tool for assessing clinical reasoning skills in general practice.

KEY MESSAGES

- The SCT is a validated and reliable tool for assessing postgraduate training in general practice.
- Clinical reasoning progresses throughout postgraduate training in general practice.
- The SCT can be used to harmonize general practice training across training sites.

## Introduction

The objective of the initial training of medical students is for them to acquire medical skills. To achieve this, they must master three areas: theoretical knowledge, professional skills and clinical reasoning. Professional skills and clinical reasoning are acquired during postgraduate medical training, including practical training [1,2], which relies on setting-specific practice and training. Currently, in France, no specific evaluation is performed to assess students’ clinical reasoning, despite the fact that this is a crucial aspect of medical training that enables clinicians to treat the information gathered in clinical situations [3].

Formative assessment plays a key role in the acquisition of medical knowledge [4,5]. It helps learning by allowing students to determine their strengths and weaknesses helping them to improve their learning. Therefore, it seems important to assess the core competencies required for the specialty in question to enable the assessment of students’ progress during their clinical practice sessions [6,7]. A new aim in Europe is to harmonize practice and learning for medical students [8]. Each country uses different tests to try to assess the clinical skills of the students. The objective structured clinical examination (OSCE) seems to be the closest test to the ideal assessment of clinical skills [9,10]. In France, it has been used in some places in a formative way but it is not used in a sanctioning way because it is not reproducible on large samples and depends on the operators. In this context, certain researchers have examined tests assessing the overall medicals skills of general practitioners (GPs) [11,12].

The script concordance test (SCT) was developed in Canada around 15 years ago and is used to assess students’ clinical reasoning [13]. This tool reflects the extent to which the candidates’ judgements map to those of a reference panel for the specialty in question in cases where there is clinical uncertainty [14]. The SCT offers a standardized assessment of the reasoning process applied to ill-defined clinical cases and has proven ability to differentiate between students and experts in relevant disciplines [15–18]. The SCT is, therefore, used to assess students’ ability to reason through complex problems that cannot be solved merely by applying knowledge [19]. Any divergence between a student’s response and that of the experts will allow the areas in which the student requires further training to be identified.

A previous study in the context of general medicine has compared the SCT with clinical reasoning problems (CRPs) but not as a valid tool for the evaluation of clinical reasoning [20]. Having a standardized tool that allows the identification students who are experiencing difficulties in acquiring clinical reasoning skills that is usable in a large sample could help to improve teaching, and adapt and hence standardize, practices.

The aim of this study was to demonstrate the reliability and validity of the SCT in the general practice context, and to assess whether this tool could be used to assess medical students’ progress in acquiring clinical reasoning.

## Methods

This study was a longitudinal observational study from November 2010 to November 2013. We first assessed the group of students at the beginning of their postgraduate training in general medicine in November 2010. They then took the same test each semester throughout the three years of their training, before their change in clinical placement. The students were given 20 minutes training on how to complete the SCT before their first assessment. We acquired data for seven sessions (S0–S6). The first session (S0) was obtained before the beginning of the students’ postgraduate medical training. S1 to S6 were collected at the end of each clinical training semester.

### The SCT

The SCT presents students with a series of uncertain clinical scenarios. Once the basic scenario has been introduced, three pieces of additional clinical information (items) are given, separately from one another. Students must then make decisions on the diagnosis, investigation and treatment for each of the three pieces of information offered, including answering three questions on a five-point Likert scale (Table 1) [21].

**Table 1.** Sample of an SCT question.

Case 1: Cindy, 28 years old, complained of nausea and faintness, for one week. On request, she signals slight breast pain and mild epigastric pain.						
And if your assumption was…	And you discover that…	The hypothesis becomes…				
1. Early pregnancy	She has an IUD and her last period was two weeks ago	–2	–1	0	+1	+2
2. Gastroenteritis	She presented with two episodes of profuse diarrhoea during the week	–2	–1	0	+1	+2
3. Latent anxiety	She has a history of depression and anorexia	–2	–1	0	+1	+2
–2: Much less likely diagnostic hypothesis (assumption virtually eliminated) –1: A less likely hypothesis	0: No effect on the diagnostic hypothesis	1: A more likely diagnostic hypothesis 2: A much more likely hypothesis (almost certainly correct)				

We used the 45-SCT developed by the Department of General Medicine at the University of Liège, Belgium. This SCT was developed as a basis for admitting students to the general medicine course. Each scenario contains three questions, giving 135 items spread across 21 diagnosis scripts (63 items), 12 investigation scripts (36 items) and 12 treatment scripts (36 items). The scenarios covered a vast number of the fields involved in general medicine (Table 2). We needed to modify four sentences to clarify them for our students.

**Table 2.** Numbers of questions in the SCT, by field and pathology.

	Pathology	Number of questions
Diagnosis	1-Gynaecology	10
	2-Gastroenterology	5
	3-Psychiatry	3
	4-Orthopaedics	11
	5-Pneumology	5
	6-Cardiovascular	8
	7-Neurology	3
	8-Ophthalmology	1
	9-Infectious	9
	10-Urology	4
	11-Oncology	2
	12-ORL	2
Investigation	1-Neurology	3
	2-Rheumatology	9
	3-Oncology	7
	4-Infectious	9
	5-Endocrinology	4
	6-Psychiatry	1
	7-Cardiovascular	1
	8-Gastroenterology	2
Treatment	1-Rheumatology	6
	2-Infectious	14
	3-Gastroenterology	1
	4-Pneumology	6
	5-Gynaecology	6
	6-Oncology	3

### Construction of the reference panel

It is recommended that 15–20 experts be assembled as a reference panel to achieve stable scores independent of the composition of the panel [14]. In this study, experts were defined as persons with broad educational or organizational responsibilities in medicine, including but also beyond, the practice level (e.g. at university or national levels). The reference panel was made up of 20 academic experts in general medicine. They took the same SCT as the students, to enable the establishment of benchmark scores. The experts took the test once, one month before the students’ first assessment.

### Administration of the SCT

Sessions S0, S1, S2, S3 and S6 were held in a classroom and under supervision. The question sheets were collected at the end of each test, and there was no time limit for the responses. The students were asked to respond using tables commonly used for multiple-choice question (MCQ) responses, such that each potential response on the five-point Likert scale corresponded to a possible response to a MCQ item. This method allowed us to use an optical reader to score the responses electronically. The results were recorded in tables that were processed by an optical reader, resulting in an Excel file.

For S4 and S5, we used an intranet platform provided by the medical faculty’s continuing education division, which allowed us to use their online SCT platform. Several studies have demonstrated the validity of online SCTs and we wished to use an online process to verify its usability [22–24].

### Scoring

The scoring system is designed to gauge the extent to which the candidate’s script matches, or is similar to, that of some experienced doctors on a reference panel [1,19,25]. For each question, the number of points awarded to the examinees for each possible response depended on the number of experts who gave the same response. The global score was obtained by adding the scores for each question and transforming this into a 100-point scale [13]. For score calculation, we used the combined scoring method described by Charlin et al. [26], as well as the calculator made available by the University of Montreal’s School of Medicine, Canada (cpass.umontreal.ca), explicitly dedicated to SCT.

### Statistical analysis

To describe the student’s sample, we used the mean, the standard deviation (SD) and standard error of measurement (SEM) through the calculation of the confidence interval (95%CI). The reliability of the test was estimated by Cronbach’s alpha coefficient for each session. For the validity, these scores were compared with experts using either a *t*-test or paired Wilcoxon test, depending on variable normality, and *p* <.05 was considered statistically significant. STATA 12 software was employed for the statistical analysis.

To evaluate the students’ progress in clinical reasoning, we determined the difference in point scores between two successive sessions for each student. We then averaged this point difference for each session, which enabled us to compare the averages every six months.

## Results

**Figure 1.**
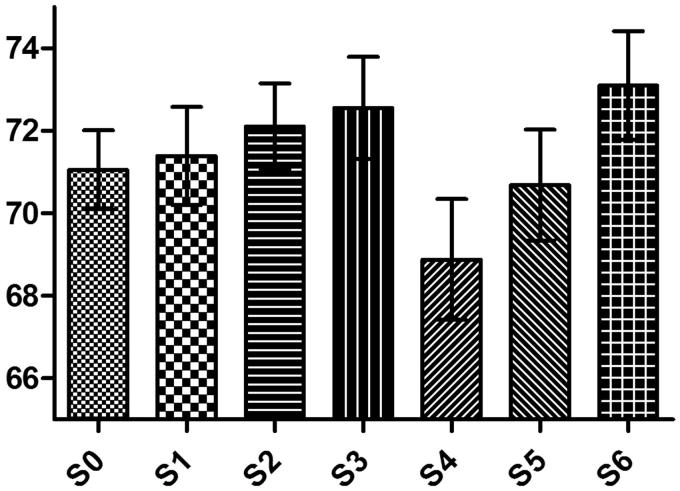
Students’ means scores and their 95%CI at each session of the test.

Ninety students from the total cohort of 135 completed all sessions (66%). The average expert score was 76.7%. The students’ scores were lower than that of the experts in all sessions, and this was statistically significant for each session (*p* <.05) (Table 3). The students’ mean scores scored ranged between 68.9 and 73.1, which was consistently lower than the experts’ mean score of 76.7. Moreover, all Cronbach’s alpha reliability coefficient values were higher than 0.6 (0.65–0.83). Individual confidence interval (95%CI individual) is around 5.5, meaning that individual score has a low zone of uncertainty (Figure 1).

**Table 3.** Students’ results on the overall test (*n* = 90).

Sessions (S)	Means (SD)	95%CI sample	Range	SEM	95%CI individual	Cronbach’s α
S0^a^	71.1 (4.5)	70.2–72.0	54.2–79.4	2.66	5.2	0.65
S1^a^	71.4 (5.7)	70.2–72.6	43.3–80.4	2.85	5.6	0.74
S2^a^	72.1 (5.0)	71.1–73.1	53.2–82.6	2.87	5.6	0.67
S3^a^	72.6 (5.9)	71.4–73.8	47.3–81.4	2.76	5.4	0.78
S4^a^	68.9 (7.0)	67.5–70.3	47.6–79.6	2.88	5.6	0.83
S5^a^	70.7 (6.4)	69.4–72.0	52.4–85.6	2.63	5.2	0.83
S6^a^	73.1 (6.3)	71.8–74.4	48.6–85.4	2.81	5.5	0.80

^a^ *t*-Test using the expert mean = 76.7; *p* value <.000.

The test was feasible either on paper or the internet SCT platform. However, we noted an average decrease in the students’ scores when they took the SCT on the internet platform. Students’ clinical reasoning progressed over the course of their three years of training, particularly during the first 18 months (Table 4).

**Table 4.** Students’ average improvement between semesters on the global test.

Sessions (S)^a^	Average improvement (SD)	95%CI	Min–max
S0–S3	1.5 (5.1)	0.5–2.0	–15.3 to 10.3
S3–S6	0.5 (6.1)	–0.75–1.75	–25.0 to 27.0
S0–S6	2.0 (6.3)	0.7–3.3	–26.1 to 14.4

^a^ Wilcoxon: S0 versus S3: *p* value <.003; S3 versus S6: *p* value <.12; S0 versus S6: *p* value <.0001.

## Discussion

### Main findings

This study used the SCT to evaluate the clinical reasoning of postgraduate general practice students. We confirmed the reliability and the validity of the SCT, individually and for the sample, to assess clinical reasoning in general practice, and show that students’ clinical reasoning changed over the 18-month clinical training period.

### Strengths and limitations

The SCT has been demonstrated to be well accepted by students, whatever their level. This probably is because it is similar to clinical procedures in real life [27]. In our study, the students, all from the University of Toulouse, took the SCT for the first time, and despite a brief introduction to the method, they did not know how to use the test. However, they appeared to be well prepared for this type of test, and there were few missing answers, despite that the test results had no impact on their overall education.

Our study involved a significant number of participants: 90 students every semester, at one university. To the best of our knowledge, only one other study has included a larger number of students (*n* = 202), bringing together postgraduate students in surgery from nine universities [28]. The participation rate in our study was high but since it was conducted at a single institution it may have generalized our results. However, we have a good sample size and low confidence interval (CI) that allows us to reflect our results in the general postgraduate students’ population.

The SCT we used was reliable: it comprised 135 clinical articles, against the minimum of 60 recommended in the literature and 20 experts in the reference panel, against the suggested minimum of 15 [21,29,30]. In addition, the reliability measure, Cronbach’s alpha coefficient, was greater than 0.6 on the test set [26]. We also noted a significant difference between the students and the experts, confirming that the SCT is capable of distinguishing between them. This has also been reported in a number of comparative studies [15–18]. We could not compare the SCT to another test because there was no gold standard to evaluate clinical reasoning. The literature described OSCE but it could not be applied to large cohorts such as ours [31]. Thus, we have a reliable and valid tool to assess the development of clinical reasoning in our postgraduate general practice students. To increase the efficiency of the test we must now broaden the themes of the scenarios, as currently it represents only part of the discipline, as well as remove indiscriminate scenarios.

### Implications

Interestingly, the results differed by approximately three points from a total of 100 when students were assessed using a paper-based test in the classroom and when they took the online SCT, although progression was still evident, and their scores returned to a constant average by the end of the assessments. We cannot explain this phenomenon since other studies have shown the online SCT to be reliable [22–24]. It is possible that the students did not concentrate on the test when taking it at home as well as they might in a classroom setting, and that this influenced the results. We chose to use the online platform for logistical reasons, thinking that it would be easier to assess large numbers of students in this way and because some were working in remote areas. Moreover, we hoped to use the SCT as a self-assessment tool in the future. However, ultimately it proved simpler and more effective to use a paper-based test and score it with an optical reader than to assess students with the online SCT test. This practical dimension needs to be considered when integrating SCTs into a general medicine training programme.

If we examine the development of clinical reasoning during postgraduate general practice training, we note that the largest increase occurs during the first 18 months (1.5 versus 0.5 points). This is because during their postgraduate training, students are implementing the scripts they have built during their undergraduate years, including during their practical training. This progression in clinical reasoning seems logical and has been shown in different populations by comparing the scores between undergraduate students, postgraduate students and experts but never in the same sample over such an extended study period. One previous study reassessed postgraduate general practice students after three months but did not show any change in their scores [20]. Hence, developing clinical reasoning is a process that takes time. This also raises the question of memorizing and learning through repetition, since our test was repeated seven times. However, the stability of the results, with their slow linear progression, as well as the fact that there were no ‘right’ answers leads us to conclude that a learning effect was unlikely.

With additional analysis, the SCT is a tool that could be integrated into the curriculum to allow the identification of students in difficulty and those that progress slowly, and enable the development of appropriate techniques to help them. To achieve this we must foresee a training programme that uses this technique and offers different methods of learning clinical reasoning [32,33]. Our study will allow us to conduct a second analysis to assess the impact of various clinical courses as students change course every six months during their clinical training. This will enable us to better tailor courses for students in difficulty. In the future, we could improve our test by removing or replacing the least discriminating items.

At the end of a students’ training, their teachers must certify whether they are competent professionals [12]. To do this, it is necessary to assess their theoretical and practical knowledge and skills. Only the SCT provides an overarching assessment of these skills and allows a comprehensive evaluation of the different facets of professional training [2]. If the evaluation by SCT becomes one of the key tools in medical education, it could be a tool that would enable the standardization of the teaching and evaluation of students in general practice on a large scale, as is desirable for the future course of our speciality [9].

## Conclusion

The SCT developed for this study was found to be reliable and capable of discriminating between postgraduate students and experts in general practice. These results demonstrate that the SCT is a useful tool for assessing clinical reasoning in postgraduate students of general practice. With additional analysis, we could propose this tool for monitoring progress in the development of clinical reasoning.

## References

[1] Charlin B, Boshuizen HPA, Custers EJ, et al Scripts and clinical reasoning. Med Educ. 2007;41:1178–1184. 18045370 10.1111/j.1365-2923.2007.02924.x

[2] Fernandez N, Dory V, Ste-Marie L-G, et al Varying conceptions of competence: an analysis of how health sciences educators define competence. Med Educ. 2012;46:357–365. 22429171 10.1111/j.1365-2923.2011.04183.x

[3] Schmidt HG, Rikers RMJP. How expertise develops in medicine: knowledge encapsulation and illness script formation. Med Educ. 2007;41:1133–1139. 18004989 10.1111/j.1365-2923.2007.02915.x

[4] Crossley J, Humphris G, Jolly B. Assessing health professionals. Med Educ. 2002;36:800–804. 12354241 10.1046/j.1365-2923.2002.01294.x

[5] Wass V, Van der Vleuten C, Shatzer J, et al Assessment of clinical competence. Lancet. 2001;357:945–949. 11289364 10.1016/S0140-6736(00)04221-5

[6] Van der Vleuten CPM, Schuwirth LWT, Scheele F, et al The assessment of professional competence: building blocks for theory development. Best Pract Res Clin Obstet Gynaecol. 2010;24:703–719. 20510653 10.1016/j.bpobgyn.2010.04.001

[7] Govaerts MJB, van der Vleuten CPM, Schuwirth LWT, et al Broadening perspectives on clinical performance assessment: rethinking the nature of in-training assessment. Adv Health Sci Educ Theory Pract. 2007;12:239–260. 17096207 10.1007/s10459-006-9043-1

[8] Windak A Harmonizing general practice teachers' development across Europe. Eur J Gen Pract. 2012;18:77–78. 22591058 10.3109/13814788.2012.684766

[9] Bouhuijs PA, van der Vleuten CP, van Luyk SJ. The OSCE as a part of a systematic skills training approach. Med Teach. 1987;9:183–191. 3670002 10.3109/01421598709089933

[10] Harden RM. What is an OSCE? Med Teach. 1988;10:19–22. 3221760 10.3109/01421598809019321

[11] Hummers-Pradier E, Beyer M, Chevallier P, et al Series: The research agenda for general practice/family medicine and primary health care in Europe. Part 4. Results: specific problem solving skills. Eur J Gen Pract. 2010;16:174–181. 20825274 10.3109/13814788.2010.504982

[12] Vermeulen MI, Tromp F, Zuithoff NPA, et al A competency based selection procedure for Dutch postgraduate GP training: a pilot study on validity and reliability. Eur J Gen Pract. 2014;20:307–313. 24645788 10.3109/13814788.2014.885013

[13] Charlin B, Roy L, Brailovsky C, et al The script concordance test: a tool to assess the reflective clinician. Teach Learn Med. 2000;12:189–195. 11273368 10.1207/S15328015TLM1204_5

[14] Gagnon R, Charlin B, Coletti M, et al Assessment in the context of uncertainty: how many members are needed on the panel of reference of a script concordance test? Med Educ. 2005;39:284–291. 15733164 10.1111/j.1365-2929.2005.02092.x

[15] Ducos G, Lejus C, Sztark F, et al The script concordance test in anesthesiology: Validation of a new tool for assessing clinical reasoning. Anaesth Crit Care Pain Med. 2015;34:11–15. 25829309 10.1016/j.accpm.2014.11.001

[16] Brazeau-Lamontagne L, Charlin B, Gagnon R, et al Measurement of perception and interpretation skills during radiology training: utility of the script concordance approach. Med Teach. 2004;26:326–332. 15203845 10.1080/01421590410001679000

[17] Iravani K, Amini M, Doostkam A, et al The validity and reliability of script concordance test in otolaryngology residency training. J Adv Med Educ Prof. 2016;4:93–96. 27104204 PMC4827762

[18] Lubarsky S, Chalk C, Kazitani D, et al The script concordance test: a new tool assessing clinical judgement in neurology. Can J Neurol Sci. 2009;36:326–331. 19534333 10.1017/s031716710000706x

[19] Lubarsky S, Charlin B, Cook DA, et al Script concordance testing: a review of published validity evidence. Med Educ. 2011;45:329–338. 21401680 10.1111/j.1365-2923.2010.03863.x

[20] Dory V, Charlin B, Vanpee D. Multifaceted assessment in a family medicine clerkship: a pilot study. Fam Med. 2014;46:755–760. 25646825

[21] Fournier JP, Demeester A, Charlin B. Script concordance tests: guidelines for construction. BMC Med Inform Decis Mak. 2008;8:18. 18460199 10.1186/1472-6947-8-18 PMC2427021

[22] Kania RE, Verillaud B, Tran H, et al Online script concordance test for clinical reasoning assessment in otorhinolaryngology: the association between performance and clinical experience. Arch Otolaryngol Head Neck Surg. 2011;137:751–755. 21844407 10.1001/archoto.2011.106

[23] Mathieu S, Couderc M, Glace B, et al Construction and utilization of a script concordance test as an assessment tool for DCEM3 (5th year) medical students in rheumatology. BMC Med Educ. 2013;13:166. 24330600 10.1186/1472-6920-13-166 PMC3878954

[24] Sibert L, Darmoni SJ, Dahamna B, et al On line clinical reasoning assessment with Script Concordance test in urology: results of a French pilot study. BMC Med Educ. 2006;6:45. 16938134 10.1186/1472-6920-6-45 PMC1574298

[25] Charlin B, van der Vleuten C. Standardized assessment of reasoning in contexts of uncertainty: the script concordance approach. Eval Health Prof. 2004;27:304–319. 15312287 10.1177/0163278704267043

[26] Charlin B, Gagnon R, Lubarsky S, et al Assessment in the context of uncertainty using the script concordance test: more meaning for scores. Teach Learn Med. 2010;22:180–186. 20563937 10.1080/10401334.2010.488197

[27] Sibert L, Charlin B, Gagnon R, et al Evaluation of clinical reasoning in urology: contribution of the script concordance test. Prog Urol. 2001;11:1213–1219. 11859654

[28] Nouh T, Boutros M, Gagnon R, et al The script concordance test as a measure of clinical reasoning: a national validation study. Am J Surg. 2012;203:530–534. 22450028 10.1016/j.amjsurg.2011.11.006

[29] Gagnon R, Charlin B, Lambert C, et al Script concordance testing: more cases or more questions? Adv Health Sci Educ Theory Pract. 2009;14:367–375. 18481187 10.1007/s10459-008-9120-8

[30] Charlin B, Gagnon R, Sauvé E, et al Composition of the panel of reference for concordance tests: do teaching functions have an impact on examinees’ ranks and absolute scores? Med Teach. 2007;29:49–53. 17538834 10.1080/01421590601032427

[31] Schoenmakers B, Wens J. The objective structured clinical examination revisited for postgraduate trainees in general practice. Int J Med Educ. 2014;5:45–50. 25341211 10.5116/ijme.52eb.f882 PMC4224044

[32] Švab I, Allen J, Žebiene E, et al. Training experts in family medicine teaching. Eur J Gen Pract. 2016;22:58–63. 26800044 10.3109/13814788.2015.1118456

[33] Kersnik J. Learning and teaching to educate future GPs. Eur J Gen Pract. 2012;18:199–200. 23205965 10.3109/13814788.2012.742060

